# Effect of different planting pattern arrangements on soil organic matter and soil nitrogen content under a maize/soybean strip relay intercropping system

**DOI:** 10.3389/fpls.2022.995750

**Published:** 2022-12-15

**Authors:** Xiao Te, Muhammad Jawad Hassan, Kuoshu Cui, Jiahui Xiao, Muhammad Naveed Aslam, Amjad Saeed, Wenyu Yang, Safdar Ali

**Affiliations:** ^1^ College of Agronomy, Sichuan Agricultural University, Chengdu, China; ^2^ Institute of Chinese Medicine Resources and Cultivation, Sichuan Academy of Traditional Chinese Medicine Sciences, Chengdu, China; ^3^ College of Grassland Science and Technology, Sichuan Agricultural University, Chengdu, China; ^4^ Dry Grain Department, Sichuan General Popularization Centre of Agricultural Technique, Chengdu, China; ^5^ College of Landscape Architecture, Sichuan Agricultural University, Chengdu, China; ^6^ Department of Agronomy, University of Agriculture, Faisalabad, Pakistan; ^7^ Department of Agronomy, The Islamia University of Bahawalpur, Punjab, Pakistan; ^8^ Department of Agronomy, Pir Mehr Ali Shah Arid Agriculture University, Rawalpindi, Pakistan

**Keywords:** intercropping, soil organic matter and total nitrogen, spatial distribution, maize (Zea mays L.), soybean

## Abstract

Assessing the spatial distribution of organic matter and total nitrogen in soil is essential for management and optimum utilization of fertilizers. Therefore, the present field experiment was conducted to evaluate the impact of different planting pattern arrangements on the spatial distribution of soil total nitrogen and organic matter content under a maize/soybean strip relay intercropping system. The planting was arranged in a manner such that soil sampling could be done from continuous maize/soybean relay strip intercropping (MS1), maize/soybean relay strip intercropping in rotation (MS2), traditional maize/soybean intercropping (MS3), sole maize (M), sole soybean (S), and fallow land (FL) from 2018 to 2020. The results showed significant variations for soil organic matter and total nitrogen content under different planting pattern arrangements of maize and soybean in the strip relay intercropping system. Across all systems, the highest soil organic matter (29.19 g/kg) and total nitrogen (10.19 g/kg) were recorded in MS2. In contrast, the lowest soil organic matter (1.69 g/kg) and total nitrogen (0.64 g/kg) were observed in FL. Soil organic matter and total nitrogen in MS2 increased by 186.45% and 164.06%, respectively, when compared with FL. Soil organic matter and total nitrogen in MS2 increased by 186.45% and 164.06%, respectively, when compared with FL. Furthermore, under MS2, the spatial distribution of soil organic matter was higher in both maize and soybean crop rows as compared with other cropping patterns, whereas the soil total nitrogen was higher under soybean rows as compared with maize in all other treatment. However, correlation analysis of the treatments showed variations in organic matter content. It can be concluded that different planting patterns can have varying effects on soil organic matter and total nitrogen distribution under the strip relay intercropping system. Moreover, it is recommended from this study that MS2 is a better planting pattern for the strip relay intercropping system, which can increase the spatial distribution of soil organic matter and total nitrogen, thereby improving soil fertility, C:N ratio, and crop production. This study will serve as a foundation towards the scientific usage of chemical fertilizers in agricultural sector.

## 1 Introduction

Soil organic matter and total nitrogen content are important indexes to evaluate soil fertility and soil quality. These indexes are essential sources and sinks of the global carbon cycle and have become one of the research hotspots in soil and environmental sciences (Johannes et al., 2020; Roberta et al., 2020; Wu et al., 2021). Although soil organic matter and total nitrogen content only account for a small part of the total soil volume, they play a vital role in balancing soil fertility, environmental protection, and sustainable agricultural production (Struijk et al., 2020). China feeds approximately 20% of the world’s population, with less than 9% of the world’s arable land area (Guo et al., 2010). The world’s population is increasing rapidly; therefore, it must produce more from the limited arable land to meet the needs of the growing population (Altieri, 1999; Fan et al., 2012). Strengthening the utilization of chemical fertilizers is an essential step in obtaining bumper crop yields (Hassanein et al., 2019; Mohamed et al., 2020); nevertheless, the indiscriminate use of fertilizers has led to increased soil nutrient imbalance in various regions of the world, notably many Asian countries (Zhou et al., 2017; Wang et al., 2020). In economically developed areas of China, a markedly disproportionate dose of fertilizers is being administered, i.e., averaging 339 kg/hm^2^, which is 1.29 times higher than the national average of 262 kg/hm^2^; however, in underdeveloped areas, the fertilization rate is only 178 kg/hm^2^ (Cho, 2007; Zhang and Zhang, 2008; Zhang et al., 2013).

Regarding the limited utilization of fertilizer resources, China adopted the concept of ecological agriculture in the last century (Shao et al., 2019; Tourn et al., 2019). In this regard, a lot of research on soil nutrient status has been carried out, and intercropping of different crops was one of the priorities. Intercropping is a cropping pattern in which two or more crops are cultivated simultaneously on the same piece of farmland (Godfray et al., 2010), which proves to be economically, ecologically, and socially profitable (Du et al., 2018; Gitari et al., 2020). According to statistics, the universal intercropping area is more than 1,109 hm^2^, which is about 3% of the total cultivated area (Li et al., 2007; Gautam et al., 2014). Common intercropping patterns principally comprise intercropping or relay strip intercropping, and strip intercropping refers to growing two or more crops in strips within a specific width, allowing alternate planting of different crops. The main difference between intercropping and strip intercropping lies in the fact that, in strip intercropping, different kinds of crops are not grown in a single row, but two or more rows of the same crop are grown together; one crop is formed in a “strip” and the interval is cultivated with another “strip” of crop, having a relatively fixed line number, line spacing, and strip width. Strip intercropping can make full use of limited land resources, improve the nutrient absorption and utilization efficiency of crops, and enhance soil fertility as well as soil quality (Zhang et al., 2019; Ahmed et al., 2020).

Under intercropping, soil organic matter and total nitrogen content, like other soil characteristics, have high spatial variabilities in various regions. Similarly, in China, there are significant differences in soil organic matter and nitrogen contents at different spatial locations at the same time (Liu et al., 2011; Yao et al., 2019). The changes in soil organic matter and total nitrogen content are dependent on farming practices such as fertilization, incorporation of crop residues, crop rotations, soil utilization, and tillage method (Huang et al., 2007). The tillage method has a great influence on soil organic matter and total nitrogen content. It was reported that intercropping could enhance the distribution and content of soil organic matter and total nitrogen (Leonard et al., 2012). Cereal/legume intercropping is widely recognized as a sustainable agricultural production system, as it can improve the symbiotic nitrogen fixation of legumes and reduce the input of chemical fertilizers (Yao et al., 2019). Intercropping of maize and soybean represents a new cereal/legume pattern, which farmers are rapidly adopting in Southwest China (Yao et al., 2019). However, there is no available literature on soil organic matter and total nitrogen spatial distribution in the maize/soybean strip relay intercropping system so far. Therefore, the objectives of the present study were to identify better management practices that could optimize land use efficiency; to understand the mechanism underlying the increased soil fertility under maize/soybean relay strip intercropping systems, especially Southwest China and similar areas; and, to quantify the relationship between soil organic matter, total nitrogen content, and planting patterns in a maize/soybean strip relay intercropping system. The results will serve as a foundation for the scientific usage of chemical fertilizers in the maize/soybean relay strip intercropping system.

## 2 Materials and methods

### 2.1 Experimental location

**Figure 1:**
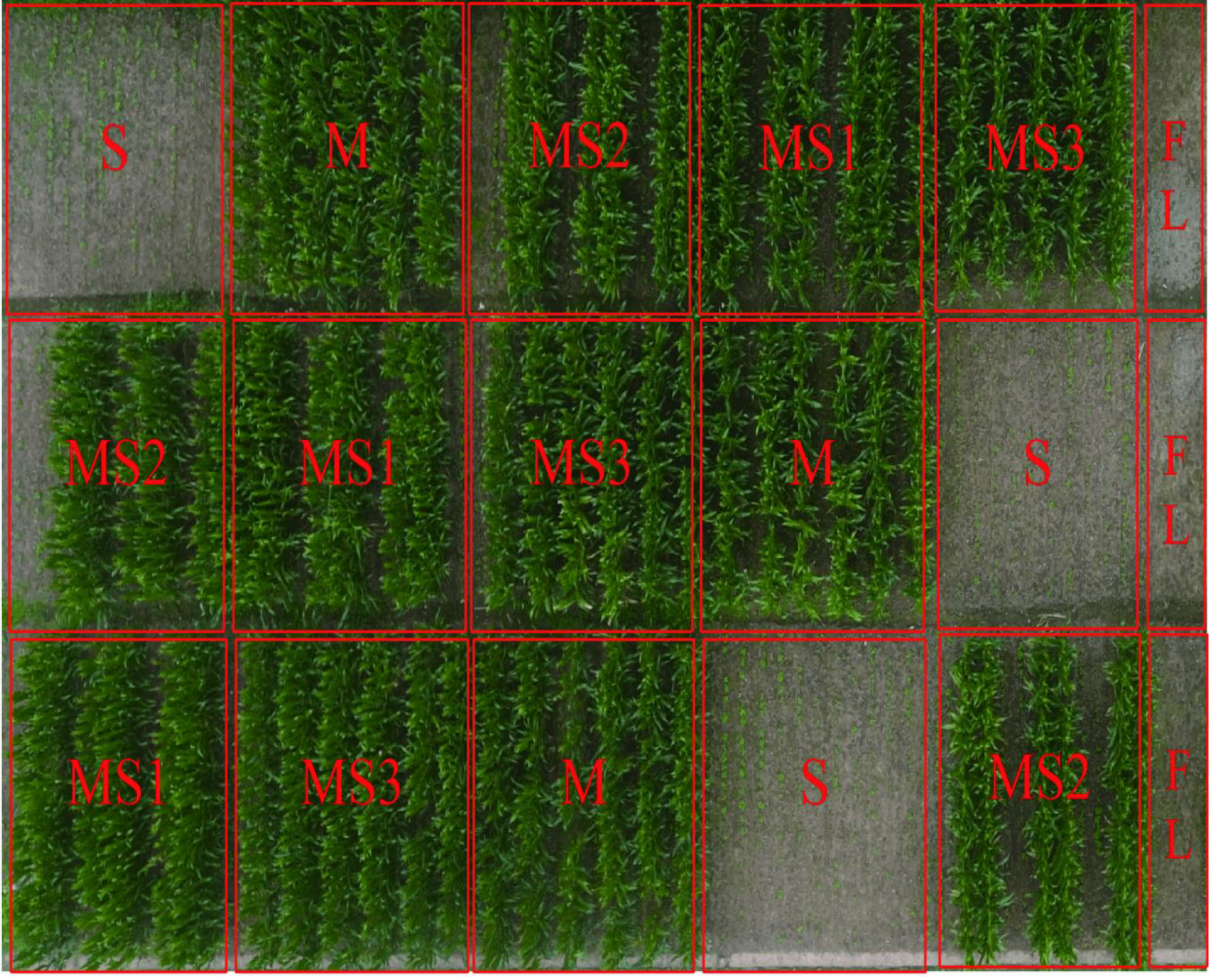
Location of the research site aerial photo. Note: MS1, MS2, MS3, M, S, FL represent the Continuous planting of maize/soybean relay strip intercropping, Planting of maize/soybean relay strip inter-cropping in rotation, Traditional maize/soybean inter-cropping, Sole maize planting, Sole soybean planting, Fallow land, respectively.

Field experiments were conducted from 2018 to 2020 at the research farm of Sichuan Agricultural University in Ya’an city located in Southwest Sichuan Province of China (101°56′26″E, 28°51′10″N). This region comprised of a hilly and mountainous topography (**Figure 1**). The climate was humid subtropical monsoon, with an average annual temperature of 16.2°C, an average annual rainfall of 1,250 to 1,750 mm, an average annual sunshine duration of 1,005 hours, and an average annual frost-free period of 300 days. The soil type was gley soils according to FAO-UNESCO 1988 (Rolf and Eddy de, 2011) with pH 6.6, organic matter 29.8 g·kg^−1^, total nitrogen 1.6 g·kg^−1^, total phosphorus 1.28 g·kg^−1^, and total potassium 14.28 g·kg^−1^.

### 2.2 Experimental design and treatments

**Figure 2:**
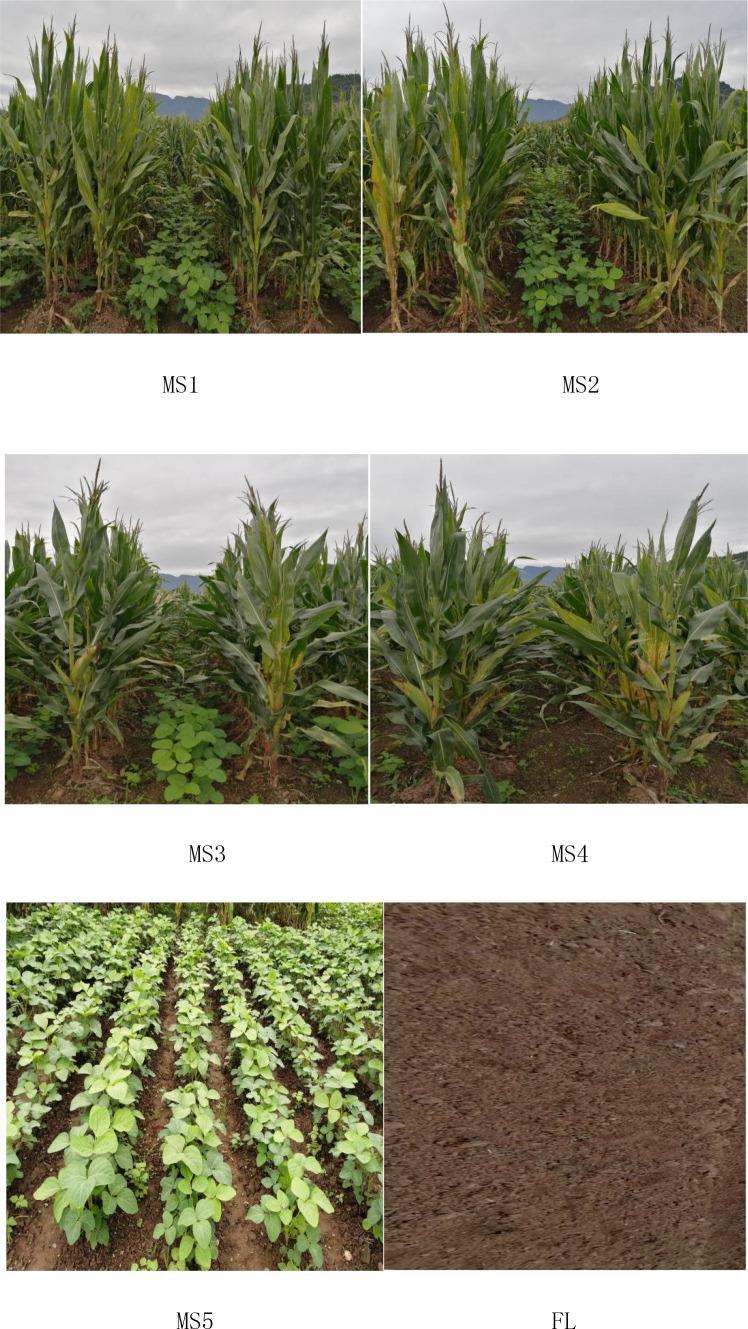
Spatial layout of different planting patterns.

The experimental design was a randomized block design with three replications (**Figure 2**). In this experiment, maize variety Denghai-605 and soybean variety Nandou-12 were used. Treatments were arranged MS1 = Continuous maize/soybean relay strip intercropping, MS2 = maize/soybean relay strip intercropping in rotations, MS3 = Traditional maize/soybean intercropping (MS3: A conventional planting method in Southwest of China), M = Sole maize, S = Sole soybean, or FL = Fallow land. The size of the experimental plots under MS1, MS2, MS3, M, and S was 6 × 6 (36 m^2^), whereas the plot size for FL was 2 × 6 (12 m^2^). The total width of MS1 and MS2 was “160 cm + 40 cm”, i.e., the relay intercropping combination of two crop strips with a total width of 200 cm, consisting of two rows of maize and two rows of soybean with a 40-cm row width for maize and soybean, and 60-cm spacing between the adjacent rows of maize and soybean. MS3 had a total width of 100 cm with a 1:1 row ratio, and distance between maize/soybean rows was 50 cm. In sole planting of maize and soybean, the distance between two rows was 100 cm for maize and 50 cm for soybean. (Note: The difference between MS2 treatment and MS1 treatment was that the maize belt and soybean belt were rotated in MS2, i.e., maize belts turned into soybean and soybean belts turned into maize each year.)

Different basal fertilizers, including urea (CH_4_N_2_O, including 46% N), calcium superphosphate [Ca(H_2_PO_4_)_2_H_2_O, including 14% P_2_O_5_], and potassium chloride (KCl, including 52% K_2_O) were used for maize and soybean. Maize was fertilized with pure nitrogen at 120 kg/ha, P_2_O_5_ at 105 kg/ha, and K_2_O at 135 kg/ha, and soybean was fertilized with pure nitrogen at 60 kg/ha, P_2_O_5_ at 63 kg/ha, and K_2_O at 52.5 kg/ha in 2018, 2019, and 2020. Maize crop was sown on 24 March, 23 March, and 29 March, in 2018, 2019, and 2020, respectively, and harvested on 25 July, 6 August, and 8 August in 2018, 2019, and 2020, respectively. Soybean was sown on 7 June, 8 June, and 13 June, in 2018, 2019, and 2020, respectively, and harvested on 30 October, 23 October, and 22 October 22 in 2018, 2019, and 2020, respectively.

### 2.3 Sample collection and measurement

**Figure 3:**
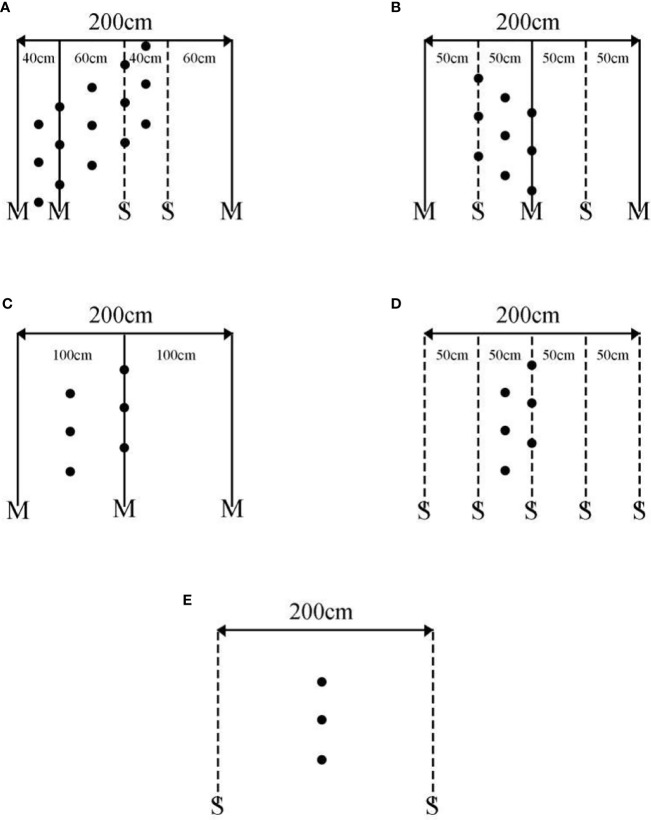
Spatial distribution of soil sample sites in different cropping patterns. **(A)** Maize/soybean relay strip intercropping (continuous and rotation cropping), **(B)** traditional maize/soybean intercropping, **(C)** maize monoculture, **(D)** soybean monoculture, and **(E)** fallow land. M, sole maize planting; S, sole soybean planting; ●, for the soil sample collection point.

In this experiment, soil samples from all the cropping patterns were collected after soybean harvesting. For soil sampling, the fixed-point sampling procedure was adopted to collect the soil sample from 0 to 20 cm soil layer (**Figure 3**). To collect the sample, a soil core was inserted vertically into the ground. All the collected soil samples were mixed, and approximately 1 kg of soil was taken for further analysis. Undisturbed soil samples were placed in a tray and stored in a clean indoor ventilation area for natural air drying. After drying, samples were put into a sample bag for the determination of soil organic matter and total nitrogen. All sample bags were labeled with a number, sampling place, soil type, sampling depth, sampling date, and time.

#### 2.3.1 Soil organic matter and total nitrogen content determination

The soil organic matter (SOM) was determined by the potassium dichromate volumetric method – external heating method (Geng and Wei, 2020).


(1), $$\text{SOM}=c\frac{({\text{v}}_{0}\text{-v)}\times 0.003\times 10724\times 1.1}{\text{m}\times k}\times 100\%$$

where c (mol/L) is the molar concentration of consuming ferrous sulfate, V_0_ is the volume (ml) of consuming ferrous sulfate in a blank test, V is the volume (ml) of consuming ferrous sulfate in the titrating soil sample, 0.003 is ¼ mmol/g of carbon, 10,724 is the conversion coefficient from soil organic carbon to organic matter, 1.1 is a correction factor (the oxidation rate in this method is 90%), m (g) is the air dry soil quality, and k is the coefficient of drying soil to drying soil.

The total nitrogen content (TN) was determined by the Kjeldahl method (Wu, 2004).


(2), $$\text{TN}=\frac{({\text{v}}_{0}\text{-v)}\times c\times 14\times 10^{-3}}{w}\times 10^{3}$$

Where V_0_ is the volume (ml) of standard acid used for titrating the sample, V is the volume (ml) of normal acid used for titrating the blank, C is the normal acid concentration (mol/L), 14 is the molar mass of N (g/mol), and W is the sample weight (g).

#### 2.3.2 Soil organic matter and total nitrogen reference standards

At present, there are many soil nutrient grading standards in China (Geng and Wei, 2020). The results of this experiment mainly refer to the national soil nutrient classification standard (**Table 1**). The Chinese soil nutrient classification standard divides soil organic matter and soil nutrient into six grades from 1 to 6. Soil organic matter and soil nutrient are the highest in grade 1 and the lowest in grade 6 (Wu, 2004). Furthermore, the spatial variation of soil organic matter and nutrient availability in China is relatively high. For example, soil organic matter in China can be as high as 200 g/kg or more, and as low as 5 g/kg or less, and the total nitrogen content can be as high as 35 g/kg and as low as 5 g/kg (Zhang et al., 2008). Therefore, further refinement of the soil organic matter and soil nutrient grade is needed to compare differences in soil nutrient grading in China (Yan et al., 2017).

**Table 1:** National soil nutrient standard grade.

Standard grade^a^	Nutrient elements	
	Organic matter (g/kg)	Total nitrogen (g/kg)
1	>40.0	>2.00
2	30.1–40.0	1.51–2.00
3	20.1–30.0	1.01–1.50
4	10.1–20.0	0.76–1.00
5	6.0–10.0	0.50–0.75
6	<6.0	<0.50

**^a^** 1, 2, 3, 4, 5, and 6 represent the first standard, second standard, third standard, fourth standard, fifth standard, sixth standard, respectively.

The classification of coefficient of variation: coefficient of variation is considered weak under <10%, moderate between 10% and 100%, and strong when it is >100% (Duan, 2000; Zhang et al., 2003).

### 2.4 Data statistics and analysis

All the experimental data were managed in Microsoft Excel 2016, and the figures were constructed with Origin Pro 2018. Differences between intercropping systems and soil organic matter and total nitrogen content were identified by analyzing variance (ANOVA) using SPSS 22.0 software (SPSS Inc., Chicago, IL, USA). The mean values were compared with a least significant difference (LSD) test at the *p* < 0.01 significance level. Linear regression techniques were used to describe the relationships between soil organic matter and total nitrogen content. The effectiveness of cropping patterns was determined by regression analysis with *p*-values (Tukey’s test) and the coefficient of determination (*R* ^2^).

## 3 Results

### 3.1 Soil organic matter content and spatial distribution

**Figure 4:**
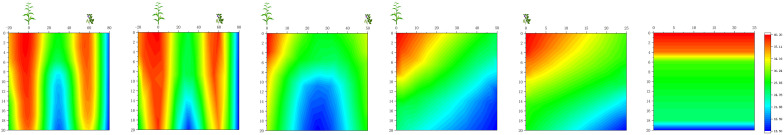
Spatial distribution of soil organic matter under different planting patterns of maize and soybean. MS1, MS2, MS3, M, S, and FL represent the continuous planting of maize/soybean relay strip intercropping, planting of maize/soybean relay strip intercropping in rotation, traditional maize/soybean intercropping, sole maize planting, sole soybean planting, and fallow land, respectively.

The different planting patterns showed significant (*p* < 0.01) variations for soil organic matter content in both maize and soybean at all sampling times across the 3 years of this experiment (**Table 2** and **Figure 4**). During the 3 years, the average SOM of each treatment order was MS2 > MS1 > MS3 > S > M > FL (**Table 2** and **Figure 4**). Furthermore, it was observed that the maximum soil organic matter (39.72 g kg^−1^) was recorded in MS2, whereas the minimum soil organic matter (8.71 g kg^−1^) was recorded in FL per system. The soil organic matter content in MS2 increased by 186.45% when compared with FL (**Table 2** and **Figure 4**). At the same time, the spatial distribution of organic matter in maize and soybean rows was most dense in MS2. The obtained results were also graded by the coefficient of variation, where we found that MS1, MS2, MS3, M, and S demonstrated moderate variations when compared, while FL showed weak variation, and overall MS2 showed the most significant variations (**Table 2**).

**Table 2:** Soil organic matter content under different planting patterns of maize and soybean in 2018–2020.

Treatment^a^	Number of samples	Content range (g/kg)	Average (g/kg)	Coefficient of variation (%)
MS1	45	15.33–36.54	27.02 ± 7.98ab^b^	29.53
MS2	45	15.51–39.72	29.19 ± 9.36a	32.06
MS3	27	14.79–34.19	25.07 ± 5.74abc	22.91
M	18	17.68–24.65	20.42 ± 2.45c	12.02
S	18	14.79–27.89	22.07 ± 3.95bc	17.90
FL	9	8.71–11.46	10.19 ± 0.71d	6.97

**^a^** MS1, MS2, MS3, M, S, and FL represent the continuous planting of maize/soybean relay strip intercropping, planting of maize/soybean relay strip intercropping in rotation, traditional maize/soybean intercropping, sole maize planting, sole soybean planting, and fallow land, respectively.

**^b^** Values followed by a different letter within the same column are significantly different at p < 0.01.

### 3.2 Total soil nitrogen content and spatial distribution

**Figure 5:**
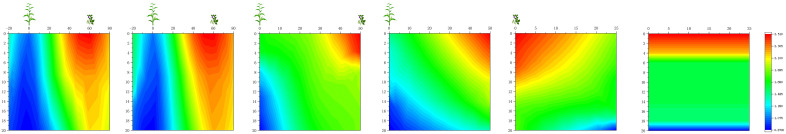
Spatial distribution of soil total nitrogen content under different planting patterns of maize and soybean. MS1, MS2, MS3, M, S, and FL represent the continuous planting of maize/soybean relay strip intercropping, planting of maize/soybean relay strip intercropping in rotation, traditional maize/soybean intercropping, sole maize planting, sole soybean planting, and fallow land, respectively.

Across all treatments, MS2 exhibited the most significant variation in total soil nitrogen content (**Table 3** and **Figure 5**). On average, the order of different treatments was MS2 > MS1 > MS3 > S > M > FL, which revealed that the minimum soil nitrogen content was under FL (0.64g/kg), and the maximum soil nitrogen content was recorded under MS2 (1.69 g/kg) (**Table 3** and **Figure 5**). However, under MS2, the spatial distribution of soil total nitrogen in soybean rows was higher when compared with maize rows (**Table 3** and **Figure 5**). The results showed that the maximum total soil nitrogen content (2.47 g kg^−1^) was recorded in MS2, while the minimum total soil nitrogen content (0.55 g kg^−1^) was recorded in S or FL, and total nitrogen in MS2 increased by 164.06% in contrast with FL (**Table 3**). Under all planting patterns, the spatial distribution of soil total nitrogen content in maize rows was lower as compared with soybean rows; however, the average maximum and minimum total nitrogen and organic matter content was almost identical under various planting patterns. Therefore, it could be speculated that organic matter and total nitrogen had a strong correlation with each other. The results showed that the MS2 planting pattern was most beneficial for soil total nitrogen accumulation.

**Table 3:** Total nitrogen content under different planting arrangements of maize and soybean in 2018–2020.

Treatment^a^	Number of samples	Content range (g/kg)	Average (g/kg)	Coefficient of variation (%)
MS1	45	0.96–2.46	1.48 ± 0.37a^b^	25.05
MS2	45	0.87–2.47	1.69 ± 0.53a	31.35
MS3	27	0.69–1.81	1.23 ± 0.29b	23.73
M	18	0.62–0.99	0.78 ± 0.13cd	16.07
S	18	0.55–1.21	0.98 ± 0.17c	17.21
FL	9	0.55–0.71	0.64 ± 0.04e	6.35

**^a^** MS1, MS2, MS3, M, S, and FL represent the continuous planting of maize/soybean relay strip intercropping, planting of maize/soybean relay strip intercropping in rotation, traditional maize/soybean intercropping, sole maize planting, sole soybean planting, and fallow land, respectively.

**^b^** Values followed by a different letter within the same column are significantly different at p < 0.01.

The correlation analysis revealed that there was moderate variation in soil total nitrogen under MS1, MS2, MS3, M, and S, and weak variation in FL; the most significant variation was observed under MS2 (*R* ^2^ = 0.96).

### 3.3 Correlation between soil organic matter and total nitrogen

**Figure 6:**
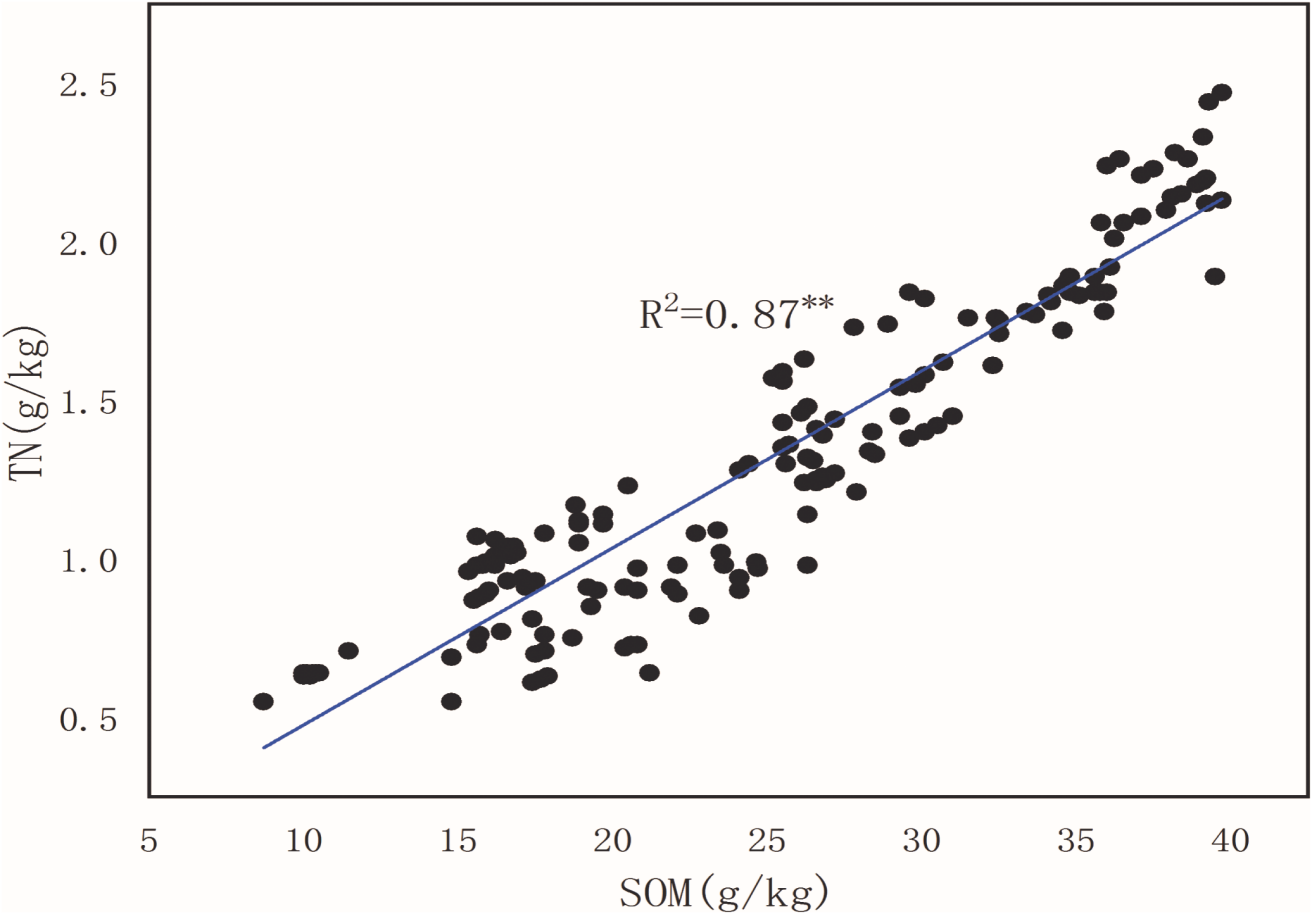
Soil organic matter (*x*) and soil total nitrogen (*y*) relationship.

Based on the results of soil organic matter and total nitrogen content in the six treated soils, the correlation equation and coefficient of determination between them could be obtained from **Table 4** and **Figure 6**. The relationship between soil organic matter (*x*) and total nitrogen (*y*) was unevenly linear. Six linear regression equations for soil organic matter and total nitrogen, and their coefficient of determinations were obtained (**Table 4**). The regression equation of the entire test area was *y* = 0.06*x* − 0.08, and the coefficient of determination was *R* ^2^ = 0.87 (**Table 4**). Furthermore, the correlation between all treatments (planting patterns) for soil organic matter and total nitrogen was significant, where MS1, MS2, MS3, and FL were closer to 1. At the same time, the soil C:N ratio was significantly different among all treatments (*p* < 0.01), and the highest C:N ratio (25.22) was recorded in MS2 (**Table 4**). These results indicated that the correlation between soil organic matter and total nitrogen in different planting methods was significant and positively correlated.

**Table 4:** Soil organic matter and total nitrogen correlation.

Treatment^a^	Sample number	Linear regression	Coefficient of determination (*R* ^2^)	Significance^b^	C:N
Whole test area	162	*y* = 0.06*x*−0.08	0.87	*p* < 0.01	19.21 ± 0.95c
MS1	45	*y* = 0.05*x*+0.25	0.96	*p* < 0.01	21.75 ± 1.07b
MS2	45	*y* = 0.06*x*+0.07	0.96	*p* < 0.01	25.22 ± 0.88a
MS3	27	*y* = 0.05*x*−0.01	0.94	*p* < 0.01	19.48 ± 1.00c
M	18	*y* = 0.04*x*−0.01	0.58	*p* < 0.01	17.30 ± 1.04d
S	18	*y* = 0.03*x+*0.25	0.60	*p* < 0.01	16.19 ± 0.87d
FL	9	*y* = 0.06*x*+0.07	0.97	*p* < 0.01	15.30 ± 0.94d

**^a^** Whole test area, MS1, MS2, MS3, M, S, and FL represent the various experiment treatments (MS1+MS2+ MS3+ M+S+ FL), continuous planting of maize/soybean relay strip intercropping, planting of maize/soybean relay strip intercropping in rotation, traditional maize/soybean intercropping, sole maize planting, sole soybean planting, and fallow land, respectively.

**^b^** Content followed by a different letter within the same column are significantly different at p < 0.01.

## 4 Discussion

### 4.1 Variations in soil organic matter content

The soil organic matter content is mainly influenced by land-use management (Demir and Ersoy, 2020; Mishra et al., 2020; Zhao et al., 2020), especially the management of different vegetation in the soil (Welegedara et al., 2020). The percentage of organic matter in shrub soil, grassland, and forest soil, within 1 m depth, was 33%, 42%, and 50%, respectively, which were significantly correlated with the type of vegetation (Eghdami et al., 2019; Yeasmin et al., 2020). Similarly, in this study, there were differences in soil organic matter content between maize and soybean planting patterns. During the 3 years, the average SOM of each treatment order was MS2 > MS1 > MS3 > S > M > FL (**Table 2** and **Figure 4**). This phenomenon might be associated with different planting patterns and crop residues of maize and soybean in the field, thereby contributing toward enhanced soil organic matter accumulation in MS2. Different land-use patterns lead to different soil cultivation, soil physical or chemical properties, and soil fertility. These variations directly affect the decomposition and transformation of soil organic matter in different soils (Lai et al., 2016; Segura et al., 2019; Liu et al., 2020). Furthermore, increased organic matter contents under different treatments in this study indicated that soil organic matter played a vital role in soil fertility under the strip relay intercropping system. Our results are consistent with the study of King et al. (2020).

### 4.2 Variations in soil total nitrogen content

The soil total nitrogen content reflects the soil potential capacity to provide nutrients for vegetation, which, together with soil organic matter and its dynamic balance, constitutes an essential index of soil fertility (Marco et al., 2011; Alhaj et al., 2019; Dai et al., 2020; Li et al., 2020; Meng, 2020). Nitrogen is considered to be blood for crop growth and development because its absorption and utilization can promote crop growth and increase the crop yield (Hua et al., 2020). Nitrogen competition is significantly higher among crop species; however, in the cereal/legumes intercropping system, it was decreased due to the nitrogen fixation mechanism of legumes, contributing toward increased nitrogen availability for absorption and utilization by cereals. Ta and Faris (1987) found that clover increased nitrogen absorption and utilization by 25% in cereals under intercropping. Broadbent (1981) reported that white clover improved the nitrogen absorption and utilization by 80% in ryegrass under intercropping. Du et al. (2019) and Raza et al. (2019) found that in the maize and soybean intercropping system, the nitrogen uptake of maize was increased by 17%–21%, which was mainly attributed to soybean nitrogen fixation. Similarly, this study demonstrated that soil total nitrogen content in maize/soybean strip intercropping was higher than sole cropping (**Table 3** and **Figure 5**). This might be related to soybean nitrogen fixation and increased total organic matter. It was found that soil nitrogen content and spatial distribution were significantly different between intercropping and monoculture, *p* < 0.01. The content and spatial distribution of soil nitrogen in the contour maps were reflected by the grading color, and the difference was obvious. Soil total nitrogen was the highest (2.47 g·kg^−1^) in MS2 treatment and the lowest (0.55 g·kg^−1^) in S and FL treatment (**Table 3** and **Figure 5**). Furthermore, this study showed that when legumes and non-legumes were intercropped, legumes’ nitrogen fixation could benefit the nitrogen absorption and uptake in non-legumes, thus promoting the growth and development of non-leguminous crops. However, the amount of nitrogen fixation depends on the different legume and non-legume intercropping combinations and different crop varieties, planting patterns, and growth habits of various crops (Gabriela et al., 2021). The biological nitrogen fixation of legumes can not only improve the nitrogen absorption and utilization of non-legumes, but can also promote growth and development, reduce crop dependence on non-renewable resources, and increase land equivalent ratio and land interest rate (Zhou et al., 2017; Chen et al., 2019). Similarly, this study demonstrated that maize/soybean relay strip intercropping has a positive effect on soil total nitrogen contents.

### 4.3 Relationship between soil organic matter and soil total nitrogen content

The soil C:N ratio not only plays a vital role in soil organic matter decomposition but is also an essential factor of soil quality evaluation, as it determines the organic matter effectiveness that improves the soil structure, enhances the carbon fixation, and increases the soil potential as a “source/sink” of atmospheric CO_2_ and nitrogen regulation (Bewket and Stroosnijder, 2003; Hagedorn et al., 2010). It is generally believed that during the initial stage of mineralization, organic matter having a C:N ratio > 30 cannot produce nitrogen. If organic matter has a C:N ratio < 15 at the beginning of mineralization, the amount of adequate nitrogen will exceed microorganism assimilation in the soil, thereby making it possible for plants to obtain adequate nitrogen from organic matter mineralization (Sun et al., 2020; Veronika et al., 2020).

Our results showed that the variation coefficient of organic matter and total nitrogen content in each treatment was moderate (**Table 4**). This might be due to the difference between organic matter and total nitrogen content. Furthermore, this could also be influenced by (1) the contrast of parent material and soil texture at the experimental site; (2) the particularity of remote terrain in the field; (3) impact of crop vegetation; and (4) climatic factors. Meanwhile, the contents of organic matter and total nitrogen in soil samples were compared, and it was found that the spatial difference of organic matter and total nitrogen was significant, and there was a significant positive correlation between organic matter and total nitrogen (**Table 4**). The results of the present study will serve as a guideline for rational fertilization in agricultural production, thereby contributing toward appropriate fertilizer usage, improved utilization rate of nutrients, and enhanced crop yield from low and medium soils.

## 5 Conclusions

Taken together, the findings of the current study elucidated the effect of different planting pattern arrangements on soil organic matter and soil nitrogen content under the maize/soybean strip relay intercropping system. It was found that different planting patterns of maize and soybean strip relay intercropping significantly affected soil organic matter and total nitrogen content in soil. The findings revealed that the highest soil organic matter and total nitrogen content was recorded in MS2, while the lowest soil organic matter and total nitrogen content was recorded in FL. Furthermore, under MS2, the spatial distribution of soil organic matter was higher in both maize and soybean crop rows as compared with other cropping patterns, whereas the soil total nitrogen was higher in soybean rows as compared with maize in all other treatments. However, correlation analysis of the treatments showed variations in organic matter content. Moreover, it is recommended that MS2 is a better planting pattern for the strip relay intercropping system, which can increase the spatial distribution of soil organic matter and total nitrogen, thereby improving the soil fertility, C:N ratio, and crop production.

## Data availability statement

The original contributions presented in the study are included in the article/supplementary material. Further inquiries can be directed to the corresponding authors.

## Author contributions

XT and WY conceived and designed the research. MH, KC, JX, MA, and AS conducted the experiments. XT and KC evaluated the data. JX and MA provided different chemical reagents and experimental material. Paper writing was completed by XT, WY, and MH. KC reviewed and edited the manuscript. All authors contributed to the article and approved the submitted version.
